# Italian Biological Prosthesis Work-Group (IBPWG): proposal for a decisional model in using biological prosthesis

**DOI:** 10.1186/1749-7922-7-34

**Published:** 2012-11-02

**Authors:** Federico Coccolini, Ferdinando Agresta, Andrea Bassi, Fausto Catena, Feliciano Crovella, Roberto Ferrara, Francesco Gossetti, Domenico Marchi, Gabriele Munegato, Paolo Negro, Micaela Piccoli, Gianluigi Melotti, Massimo Sartelli, Michele Schiano di Visconte, Mario Testini, Paolo Bertoli, Michela Giulii Capponi, Marco Lotti, Roberto Manfredi, Michele Pisano, Elia Poiasina, Eugenio Poletti, Luca Ansaloni

**Affiliations:** 1 General and Emergency Surgery Department, Ospedali Riuniti, Bergamo, Italy; 2 General Surgery Department, Adria Hospital, Rovigo, Italy; 3 General Surgery Department, Cotugno Hospital-CTO, Naples, Italy; 4 General and Emergency Surgery Department, Ospedale Maggiore, Parma, Italy; 5 General Surgery Department, Ospedale Centrale, Bolzano, Italy; 6 General Surgery Department, La Sapienza Univesity Hospital, Rome, Italy; 7 General Surgery Department, New Sant’Agostino Hospital, Modena, Italy; 8 General Surgery Department, Conegliano Hospital, Treviso, Italy; 9 General Surgery Department, Macerata Hospital, Macerata, Italy; 10 Endocrine, Digestive and Emergency Surgery Department, Aldo Moro University Hospital, Bari, Italy

**Keywords:** Biological prosthesis, Work-group, Indication, Cross-linked, Non-cross-linked, Surgery, Reconstruction, Infection, Tissue loss, Mesh

## Abstract

**Introduction:**

Indications for repair of abdominal hernia are well established and widely diffused. Controversies still exist about the indication in using the different prosthetic materials and principally about the biological ones.

**Material and methods:**

In February 2012, the Italian Biological Prosthesis Work-Group (IBPWG), counting a background of 264 biologic implants, met in Bergamo (Italy) for 1-day meeting with the aim to elaborate a decisional model on biological prosthesis use in abdominal surgery.

**Results:**

A diagram to simplify the decisional process in using biologics has been elaborated.

**Conclusion:**

The present score represents a first attempt to combine scientific knowledge and clinical expertise in order to offer precise indications about the kind of biological mesh to use.

## Introduction

Prosthetic abdominal wall surgical repair is a common procedure [1,2]. Actually, about one million prostheses per year for abdominal wall repair are used worldwide [3]. Since the first description of a mesh use for abdominal wall repairing [4] plenty of new material have been introduced, first synthetic, but later biologic. Indications for repair are well established and widely diffused [5]. However controversies still exist about the indication in using the different materials and principally about the biological ones. More than a dozen of biological prosthesis (BP) are currently available (Table 1). All of them are derived from human or mammalian tissues [6]. It has already been noted the major variability among human dermis prosthesis than among the animal ones in terms of mechanical and physical properties [6]. In fact xenograft products are obtained from a more uniform animal population with similar age and life histories, this allows producers to obtain more consistent implants than from humans donors [6].

**Table 1:** Biological prosthesis currently on the market

**Name**	**Manufacturer**	**Tissue source**	**Material**	**X-linking**
Alloderm	LifeCell	Human	Acellular dermis	No
AlloMax	Bard	Human	Acellular dermis	No
Flex HD	Ethicon/MTF^¥^	Human	Acellular dermis	-
DermaMatrix	MTF^¥^	Human	Acellular dermis	No
Permacol	Covidien	Porcine	Acellular dermis	Yes
CollaMend	Davol/Bard	Porcine	Acellular dermis	Yes
Strattice	KCI/LifeCell	Porcine	Acellular dermis	No
XenMatrix	Brennan Medical	Porcine	Acellular dermis	No
Surgisis	Cook	Porcine	Small intestine submucosa	No
Surgisis Gold	Cook	Porcine	Small intestine submucosa	No
Lyosis	Cook	Porcine	Lyophilized small intestine submucosa	No
FortaGen	Organogenesis	Porcine	Small intestine submucosa	Yes
SurgiMend	TEI bioscience	Bovine	Fetal dermis	No
Periguard	Synovis	Bovine	Pericardium	Yes
Veritas	Synovis	Bovine	Pericardium	No
Tutomesh	Tutogen	Bovine	Pericardium	No
Tutopatch	Tutogen	Bovine	Pericardium	No

^¥^ MTF: Muscoloskeletal Transplant Foundation.

BP, independently from the origin, could be further and basically divided into two main groups: cross-linked and non-cross-linked. The difference between these two groups is the proceeding the cross-linked are submitted to. The introduction of chemical cross-linking between the collagen chains, strengthens the prosthesis reducing the efficacy of bacterial and host collagenase enzymes, thus the implant is less prone to degradation in vivo [7,8].

On the basis of either the presence or not of the cross-linking, biological prosthesis are divided into two subgroups: the partially remodeling (over time) and the completely remodeling ones. The partially remodeling (cross-linked) prosthesis are made of porcine or human dermal collagen and bovine pericardium collagen [6]. The completely remodeling (not cross-linked) ones are principally made of swine intestinal sub-mucosa, swine dermis, human dermis, fetal bovine dermis and bovine pericardium. The differences in remodeling times should be kept in mind when these materials are chosen for abdominal wall repair [6]. Each type of prosthesis allows and encourages host tissue ingrowth, although different prostheses can feature different clinical attributes. Thanks to the presence of additional linkages the partially remodeling ones resist better and for a longer period to mechanical stress. Moreover BP have the lowest adhesiogenic potential among all prosthetic materials available for intra-peritoneal use [9]. Post-operative pain and discomfort have been demonstrated to be inferior when biological prosthetic materials are used in groin hernia repair [10]. Implants would act as a scaffold inside which the host tissue cells and fibroblasts can replicate. They also provide resistance to tension and stress by supporting the abdominal wall until it is fully recovered. Times of remodeling range between a few months and few years [11]. It depends on prosthesis characteristics and host tissues properties.

Surgeons have not widely assumed the capability to manage with BP. The way to consider them should be completely different from the standard synthetic meshes. These last ones are as a “patch to apply on a hole”; essentially they trigger a foreign body host response leading to encapsulation of the prosthesis with intense fibrous reaction. On the contrary BP activate a remodeling process in which the host remodels the prosthesis and his own tissues by producing new healthy tissue. By using BP the surgeon starts a real tissue engineering process [12]. The scarcity of knowledge about BP is also due to the lack of high-evidence level literature about the topic. For this reason the Italian Chapter of the European Hernia Society has founded the Italian Register of Biological Prosthesis (IRBP) to archive and study the BP use in Italy. A similar registry associated with the European Hernia Society, the European Register of Biological Prosthesis (ERBP), is currently recruiting cases all over Europe [3].

To give an answer to the many different questions about the BP use in abdominal surgery we promoted a meeting of the Italian Biological Prosthesis Work-Group (IBPWG) constituted by surgeons with a wide experience in abdominal surgery either in elective or in emergency setting and in the use of BP. The purpose of the summit was mainly to give a few simple and reproducible indications about the correct use of BP in abdominal wall surgery keeping into consideration the two main challenges: the infected fields and the loss of tissue.

## Material and methods

In February 2012, the IBPWG met in Bergamo (Italy) for 1-day summit with the aim to elaborate a decisional model on BP use in abdominal surgery. The group is constituted by general surgeons, all with extensive experience in abdominal wall surgery either in elective or in emergency setting, added to a wide experience in the use of BP (at the time of the meeting the participants had collectively implanted 284 BP).

## Results

**Figure 1:**
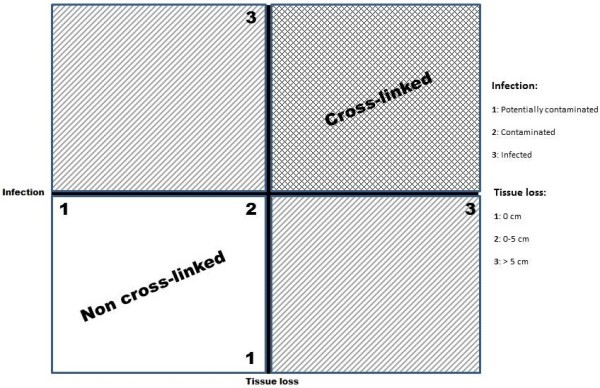
Decisional model diagram: the product of the infection and the loss of tissue scores gives as a result the value which indicate the kind of biological prosthesis to use.

**Figure 2:**
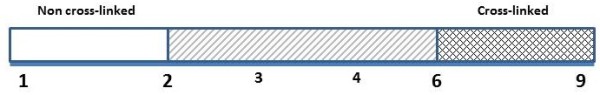
Decisional line: the different results indicate the kind of biological prosthesis to use.

A diagram to simplify the decisional process in using BP has been elaborated (Figures 1, 2). It keeps into consideration the different kind of BP, the infection of the surgical field and the tissue loss.

The diagram suggests the type of BP that should be used by combining these three variables together on the basis of scientific literature and expert opinions.

## Discussion

Complex abdominal hernia repair represents a significant challenge for surgeons. Complex hernia could be differently defined. The complexity of hernias could derive from contamination/infection, tissue loss, dimensions, anatomic position and clinical or pharmacological data.

For sure the introduction of tension-free techniques, thanks to the use of prosthetic materials, has greatly facilitated the duty. On one hand prosthetic techniques have been demonstrated to reduce the recurrence rate, on the other hand they introduced a series of new variables to take into consideration when repairing abdominal wall defects: actually prosthetic infection, dislocation, chronic pain, shrinkage, adhesions formation, fistula formation and skin erosion complicate the decision process in abdominal wall repair surgery. With the introduction of resorbable materials some of these factors have been eliminated with an increased recurrence rate as a counterpart. BP has completely changed the way to face the abdominal hernia surgery. They introduced the tissue engineering in field of the surgical practice [12]. The implant of biologic materials elicits a cascade of events leading to new healthy tissue deposition and prosthesis remodeling. It also allows to blood, growth and pro-/anti-inflammatory factors and drugs to reach the surgical field during the first phases of healing process. This for sure enhances the effect against potential or definite contamination/infection. Moreover the adhesiogenic power of BP is absolutely lower than the one of the other synthetic materials [13,14]. On the contrary there are a few doubts about the intra-peritoneal use of BP from the biomechanical point of view. It has been demonstrated that the best integration is reached if they are placed pre-peritoneally with a greater incorporation strength, less adhesion area and lower adhesion scores compared with intra-peritoneal placement [15]. Given that the long-term persistence of the prosthesis is crucial, some authors stated that the BP durability has a direct impact on the recurrence rate [16]. However durability depends on the implant intrinsic properties and also on the environment into which the BP are placed [16]. It has been demonstrated in animal models as the tensile strength is different between cross-linked and non-cross-linked meshes during the first months after the implant. However it reaches similar values after 12 months with the two kind of implants [8]. Moreover the strength of the repair sites doesn’t change over time. This might indicate that new tissue is deposited in the repair site as the scaffold is degraded, preventing the site from weakening over time [8]. Another factor that should be kept into account in choosing which kind of BP to use is the demonstration that non-cross-linked material exhibits more favourable remodeling characteristics [8]. This has a great importance when BP are used as bridging or alternatively as reinforcement. In fact discordant data have been published about the use of BP to bridge wide defects [16]. Few different non-randomized studies have been published reporting recurrence rate ranging between 100% and 0% if the prosthesis are placed respectively either as a bridge or not [16-19]. Even if high-quality comparative data about BP exist in animal models, only clinical reports of a restricted number of cases are reported for humans. Moreover only the recurrence rate is registered as outcome in almost all studies. Other data regarding the use of BP as wound classification, contamination risk/grade, associated therapy or comorbidity are seldom reported. These data are needed to completely assess the usefulness, the efficacy and the versatility of BP. All reported data derived by retrospective uncontrolled series of limited number of patients. The methodology is seldom reported and/or poorly described. Moreover the time to recurrence is rarely evaluated [16]. One last observation is that the different studies reported data about non-homogeneous cohorts of patients. Different surgical techniques, different surgeons’ skill and expertise in using BP and different hernia sites are often mixed together. These inconsistencies are probably due to the poorness of cases for each single centre. No definitive evidence based conclusions could be obtained from the literature. The majority of surgeons stated that they use BP in “difficult” situations, especially those with contaminated or infected field [16,20,21].

The present decisional model suggests, at the best of our knowledge, the way to apply scientific knowledge to the clinical practice in order to choose which type of BP use in abdominal wall defects repair. This should always be a dynamic process mediated by the surgeon decisional capability. We resumed the principal variables to keep in mind in deciding the kind of BP to use. Infection has been divided into three possible grades:

– 1: potentially contaminated

– 2: contaminated

– 3: infected

The same three steps division has been adopted for the tissue loss:

– 1: no tissue loss (only reinforcement)

– 2: 0–5 cm defect

– 3: >5 cm defect

By combining together these variables (multiplication) we obtained a score which determine the necessity to use either a cross-linked or a non-cross-linked BP (Figures 1, 2).

Operating field has been divided into three groups. In a previous grading system by the Ventral Hernia Working Group (VEWG) the four grade of risk for surgical site occurrences have been differentiated by considering also the comorbidity of the patients [5]. Clinical conditions are to be kept in mind in evaluating the use of prosthesis but in the present decisional model the principal aim is to help the surgeon to decide whether use cross-linked or non-cross-linked BP.

Undefined situations still exist. Cases with a score between 2 and 6 represent all that patients with a big tissue loss and a potential/low grade infection or vice-versa cases with an high grade infection and a low or null tissue loss. These cases need a cautious evaluation by the surgeon to establish if the priority has to be given to the tissue loss or to the grade of infection. The VEWG score could help in deciding. Infected fields with no residual loss of tissue don’t represent an absolute indication for BP use. On the contrary a small tissue loss with concomitant low/null infection but high comorbidity could suggest using a non-cross-linked BP. The higher resistance to protease enzyme action and to mechanical stress of cross-linked BP suggest using them in situation of high infection and/or big defects. As counterpart, however even in presence of a high grade infection with a low grade tissue loss could be suggested to place a non-cross-linked BP.

## Conclusion

The present score represents the first combination of scientific knowledge and clinical expertise that gives some indications about the kind of BP to use. However no definitive recommendations could be given in complicated abdominal wall reconstructive surgery. The lack of definitive evidence-based data and the high costs of the BP suggest to cautiously evaluate each single case.

## Competing interests

All authors declare no conflict of interest.

## Authors' contributions

All authors participated to the meeting in Bergamo in order to elaborate the decisional model on biological prosthesis use in abdominal surgery, proposed in this article. FeCo and LA drafted the manuscript All authors read and approved the final manuscript.
